# Simulation training program for vacuum application to improve technical skills in vacuum-assisted vaginal delivery

**DOI:** 10.1186/s12884-021-03829-y

**Published:** 2021-04-28

**Authors:** Paolo Mannella, Mario Giordano, Maria Magdalena Montt Guevara, Andrea Giannini, Eleonora Russo, Federica Pancetti, Marta Caretto, Tommaso Simoncini

**Affiliations:** Department of Clinical and Experimental Medicine, University of Pisa, Via Roma 67, 56126 Pisa, Italy

## Abstract

**Objective:**

evaluation of technical skills of the operators during the obstetrical device application for operative vaginal delivery, named kiwi-cup in a simulation training program.

**Methods:**

Thirty-five residents in obstetrics and gynecology of the University of Pisa, Italy were recruited and evaluated with an assessment scale on technical skills from 0 to 55 points. They performed various operative vaginal delivery simulations with kiwi-cup and were evaluated at time 0 by a tutor. After 8 weeks, simulation training was repeated and trainees were re-evaluated by the same tutor.

**Results:**

after 8 weeks from the first simulation session, trainees have been shown to increase technical skills (46.27 ± 4.6 with *p*-value < 0.0001), the successful application rate (85.71% with *p*-value 0.0161).) and to reduce the time to complete the procedure (86.2 ± 29.9 s with *p*-value < 0.0001).

**Conclusion:**

simulation training on operational vaginal delivery significantly increases technical skills, improves successful rate, and reduces the time taken to complete the procedure.

**Clinical trial registration:**

Not applicable.

## Précis

Simulation training program improves technical skills, successful rate and reduces time taken to complete the kiwi-cup application.

## Introduction

Operative vaginal delivery (OVD) is an obstetrical procedure used to expedite birth after full cervical dilatation when a rapid expulsion of the fetus is required. The rate of OVD changes worldwide. In the US the rate of OVD is around 3.3% [1], in the UK, OVD is performed in 15% of vaginal deliveries. In other countries, the rate is lower (between 3.4 and 5.1% in Italy) [2]. Among OVDs we recognize two types of procedures: vacuum and forceps. In the US, obstetricians prefer to use vacuum in about 82% of the cases [[3]9463].

However, these procedures require complex skills which include accurate diagnosis, understanding of the anatomy, continuous communication with colleagues, and finally the capacity to perform the correct application of the instrument.

The acquisition of the skills for vacuum application depends on the exposure of junior obstetricians to situations in which they can learn the procedure and train themselves, under the supervision of senior operators [4, 5]. The experience of trainees is therefore directly proportional to the number of operative deliveries that take place on patients in the structure where the training takes place. Obviously, this is ethically unacceptable and this teaching system was such standardized in the past that some guidelines had to insert training as “relative contraindication to execute OVD”. This is probably an emblematic and unique case in the history of medicine [[6]9448]. However, according to a recent survey carried out in Germany, even senior obstetricians still require specific and continuous training on performing OVD which obviously goes beyond just on-site professional experience [[7]9449]. For this reason, the French College of Gynaecologist and Onstetricias, supports the use of simuation for training in OVD [8].

It is important to remark that OVD is a life-saving procedure for the fetus and mother but also potentially dangerous. The main indications are the shortening of the second stage and the suspected fetal compromise. Additionally, there are other indications such as obstructed labor, maternal exhaustion, or medical condition requiring shortening of the second stage of labor which remain a major cause of maternal and neonatal mortality and morbidity across the world. Overall such complications are responsible for 4 to 13% of maternal deaths in Africa, Asia, Latin America, and the Caribbean, and obstructed labor alone accounted for 0.4 deaths per 100,000 women worldwide in 2013 [9]. Consequently, the most difficult applications, where even the experience gained would be greater, are performed only by highly qualified personnel and rarely during the training.

From an educational point of view, these elements can constitute a significant bug in the training of operators. For this reason, the Royal College of Obstetricians and Gynecologists has developed a structured training course to ensure the provision of OVD skills to the junior trainees during their training, the RCOG Operative Birth Simulation Training course, (ROBuST). In 2017, the ROBuST course was introduced as a mandatory part of the specialty training curriculum for junior obstetricians in the UK [10].

However, the UK education system is different from most European countries. Subsequently, European realities do not permit this type of training and certification.

The purpose of this study is to verify how the activation of a simulation program on the application of the vacuum, together with the creation of an evaluation scale, could help the trainees in the correct application of the vacuum.

## Material and methods

In this study, 35 residents in obstetrics and gynecology of the University of Pisa were recruited. Residents participated in a series of simulations on physiological birth, breech presentation, shoulder dystocia, and postpartum hemorrhage. The present data were obtained by extrapolating the records of 2 separate simulation sessions on OVD performed 8 weeks apart, by using “*Lucy and her Mum*” (the MODEL-med®) and all interested residents gave their consent to participate.

**Fig. 1:**
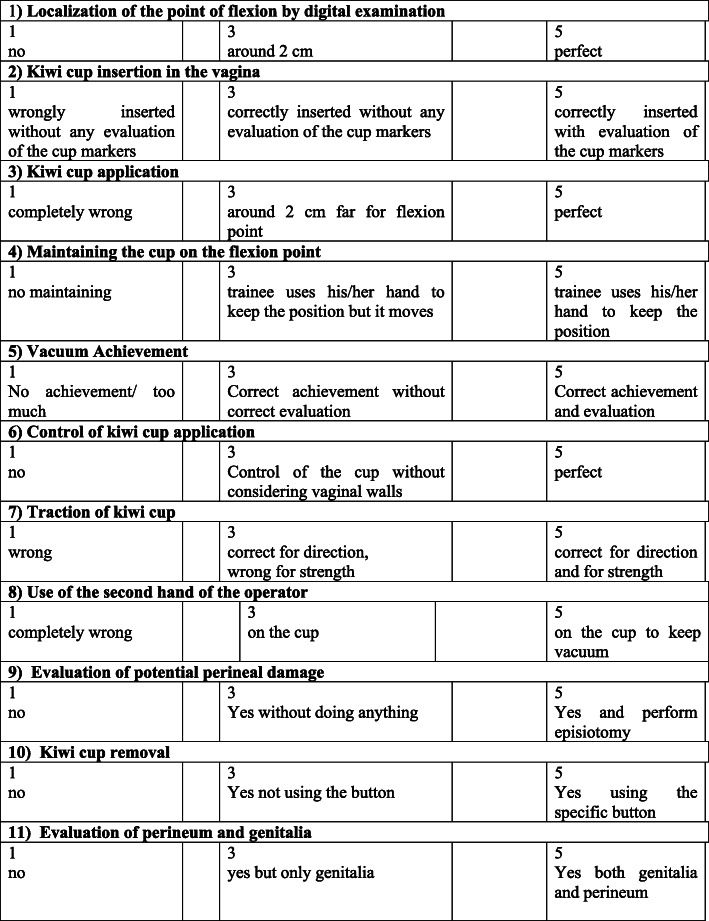
Evaluation scale for kiwi-cup application

Several sessions of simulation on OVD with different scenarios were performed (at least 5 for a session) but the evaluation of residents was performed only for a specific scenario at the beginning of sessions 1 and 2. This was decided to avoid possible errors. The first simulation on session 1 was to verify the initial background of residents and the first simulation of session 2 was to verify the effects of simulation training on OVD technical skills after 8 weeks. At the beginning of each evaluation test, residents had to fill out a self-evaluation test (Fig. 1).

In literature, there is not a standardized assessment scale to evaluate the correct application of the vacuum. Usually, it is a post-procedure evaluation: number of detaches, tractions, directions. We created a checklist considering the most important aspects to reach an optimal application of kiwi-cup (Fig. 1).

In this form, it was possible to verify the competence of the resident to perform an obstetric evaluation before using the vacuum but also to quantify the grade of stress on performing OVD with specific questions about the capacity to afford this type of situation with a rate from 1 to 5.

**Fig. 2:**
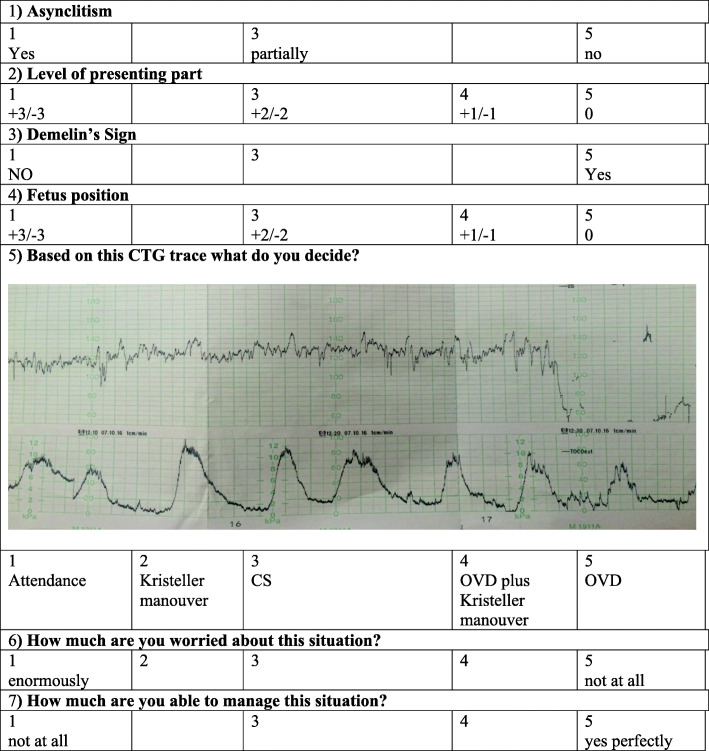
Self-assessment test: it is divided in self-evaluation queries (1–4) and self-management queries (5–7)

During the simulation test, an expert tutor valued the performance of residents with a specific tab (Fig. 2).

The resident had to follow the exact sequence in the OVD application by making the correct moves. To verify specific aspects which could be misevaluated by external observers, some tricks were used. For example, to evaluate the correct evaluation of the point of flexion or the point of kiwi-cup application, we used washable ink on the top of the gloves or of the kiwi-cup system. In this way, we evaluated if the position was correct.

The time needed to complete the operation was measured and the number of applications of the vacuum, the number of tractions needed to carry out the birth, and the ability to interact with the woman were considered.

If the resident did not perform all the necessary maneuvers, or some of them were incorrect, the scenario was not interrupted. Following Fig. 1, a tutor gave a vote for each phase from 1 to 5, where the minimum was 1 and the maximum score was 5. The whole tab is divided into 11 questions, for a total of 55 points.

After that, simulation session 1 continued with other OVD scenarios (at least 5 for each resident) to consolidate the technical skills acquired during the training.

After 8 weeks, a new simulation session was repeated (session 2). At the beginning of this session, the same scenario of the evaluation of session 1 was repeated. Residents filled out self-assessment and their performance was re-considered.

### Scenario design

Residents were instructed about the need to face the scenario as if it were a real situation. Before entering in the simulation room, each trainee received information regarding the patient: a 31-year-old woman, Caucasian, nulliparous, local anesthesia, in the second stage of labor with fetus in cephalic presentation, with no apparent risk factors.

The patient was at full cervical dilatation from around 2 h, and she started to push from 1 h. Resident was called because patient presented a pathological cardiotocographic (CTG) trace.

After the obstetric evaluation, on the basis of the CTG recording, each trainee was asked to develop the safest and fastest strategy to allow the woman to give birth, guaranteeing the safety of the fetus. The correct obstetric evaluation was: no asynclitism, level of presentation part + 1 (fetal station), Demelin sign positive [11], occiput-anterior position.

### Statistical analysis

Statistical analysis was performed using GraphPad Prism 7 (GraphPad Software). Continuous variables were presented as means ± standard deviations. D’Agostino & Pearson normality test was used to determine the normality of data distribution. A paired T-test two-tail or Wilcoxon matched-pairs signed-rank test was used to study general scores and time analysis outcomes between the first and second session. In addition, ordinary one-way ANOVA was performed followed by Tukey; multiple comparison tests were used when residents were divided into two groups: junior (1st, 2nd, 3rd year) and senior (4th, 5th year). For the improvement of the successful rate with training simulation Chi-Q Fisher’s exact test was used, and the categorical variables were presented with percentages. Values of *p* < 0.05 were considered significant (*p < 0.05; **p < 0.01; ***p < 0.001).

## Results

### Training with simulation improves technical skills

**Fig. 3:**
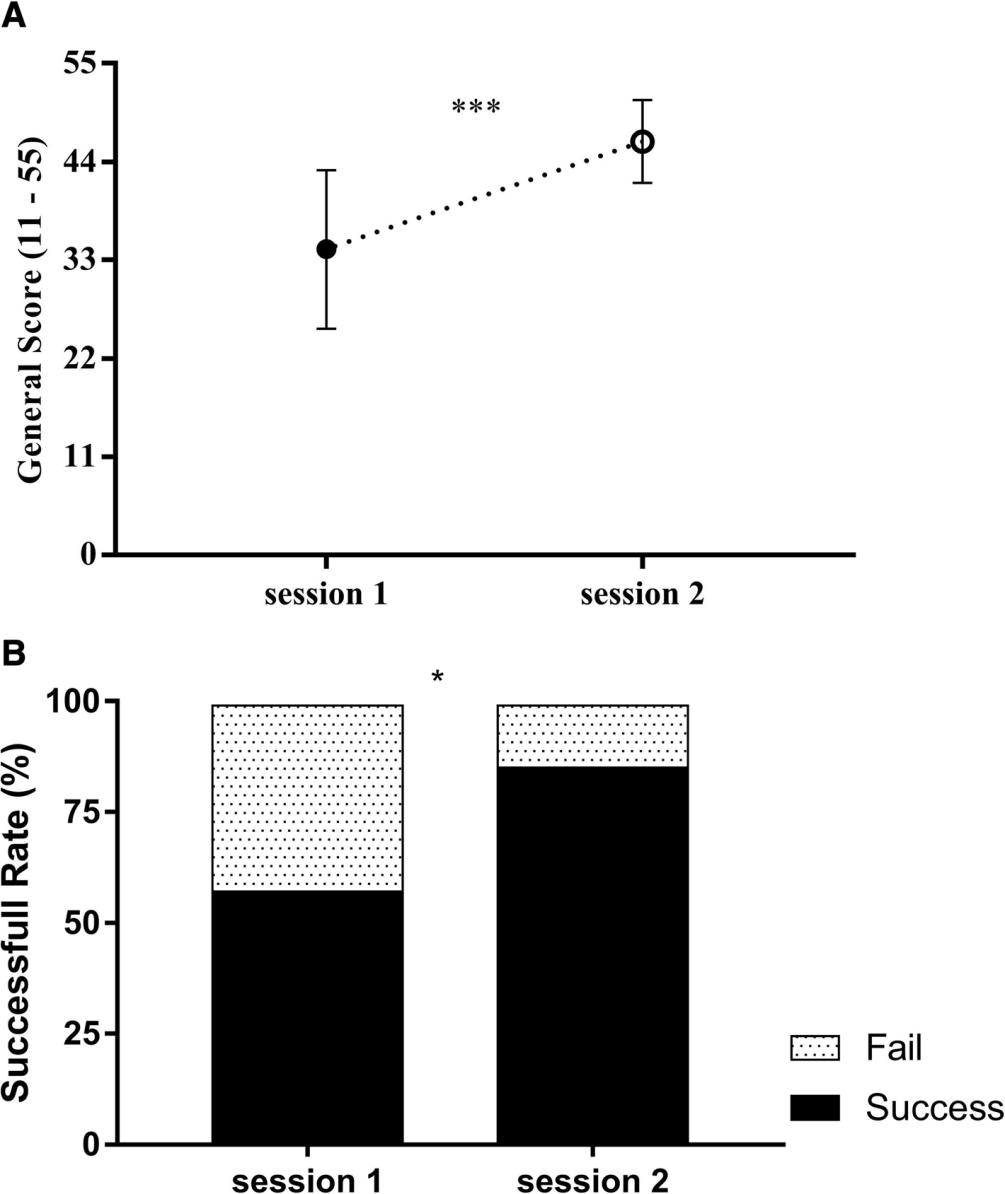
Overall performance. Training with simulation improves technical skills and successful rate. Assessments were carried out by an external observer. **a.** Training session 2 was 8 weeks later with the same scenario of session 1. Data are presented as means ± standard deviation and were analyzed statistically by paired T-test two-tail to study general scores outcomes. *p* value < 0.0001 (***). **b.** Overall successful was considered if the baby head gets out with kiwi-cup detach < 3 and within 3 contractions/pushes of the mother. Data are presented as percentages and were analyzed statistically by Chi-Q Fisher’s exact test. This successful overall was found statistically significant with a *p* value of 0.0161 (*)

The use of simulation for OVD training significantly increases general technical skills in residents (Fig. 3a). Before the training start, in session 1, the total score was 34.2 ± 8.8. After the simulation training session, 8 weeks later, in the same scenario, the total score was 46.27 ± 4.6 with a *p*-value < 0.0001.

### Training with simulation improves successful rate

Simulation improves technical skills but also the overall successful rate of OVD. We considered an OVD successful if the baby head gets out with kiwi-cup detaches less than 3 times and within 3 contractions/pushes of the mother. The use of simulation for OVD training significantly increases the successful rate (Fig. 3b). Before the training start, in session 1, the successful rate was 20 out of 35 attempts (57.14%). After the simulation training session, 8 weeks later, with the same scenario, the successful rate was 30 out of 35 attempts (85.71%). This increase was statistically significant (*p*-value 0.0161).

### Training with simulation reduces the time of vacuum application

**Fig. 4:**
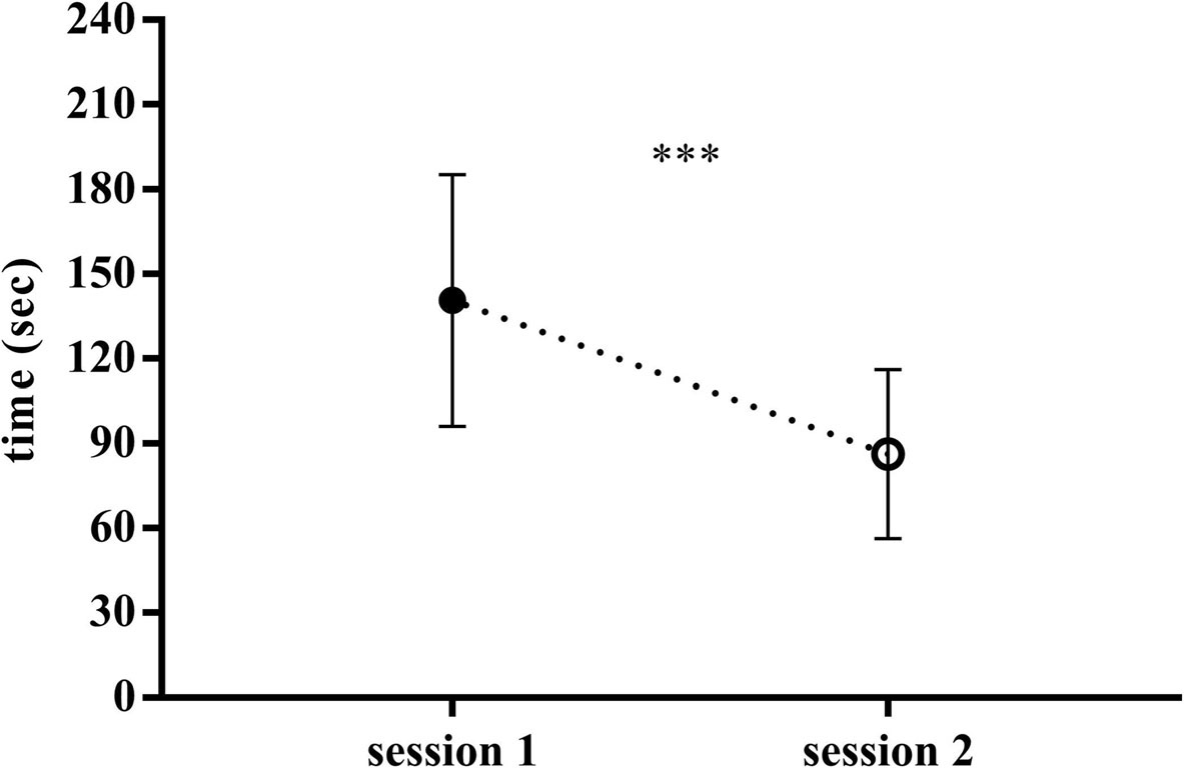
Training with simulation reduces time of vacuum application. Assessments were carried out by an external observer. Training session 2 was 8 weeks later with the same scenario of session 1. Data are presented as means ± standard deviation and were analyzed statistically by paired T-test two-tail. *p* value < 0.0001(***)

The use of simulation for OVD is important not only to increase competency in vacuum application and successful rate but also to reduce the time to complete the procedure (Fig. 4). Before the training start, in session 1, the time to complete the procedure was 140 ± 44.5 s. After the simulation training session, 8 weeks later, in the same scenario, the time to complete the procedure was 86.2 ± 29.9 s with *p*-value < 0.0001.

### Training with simulation improves technical skills both in junior and senior residents

**Fig. 5:**
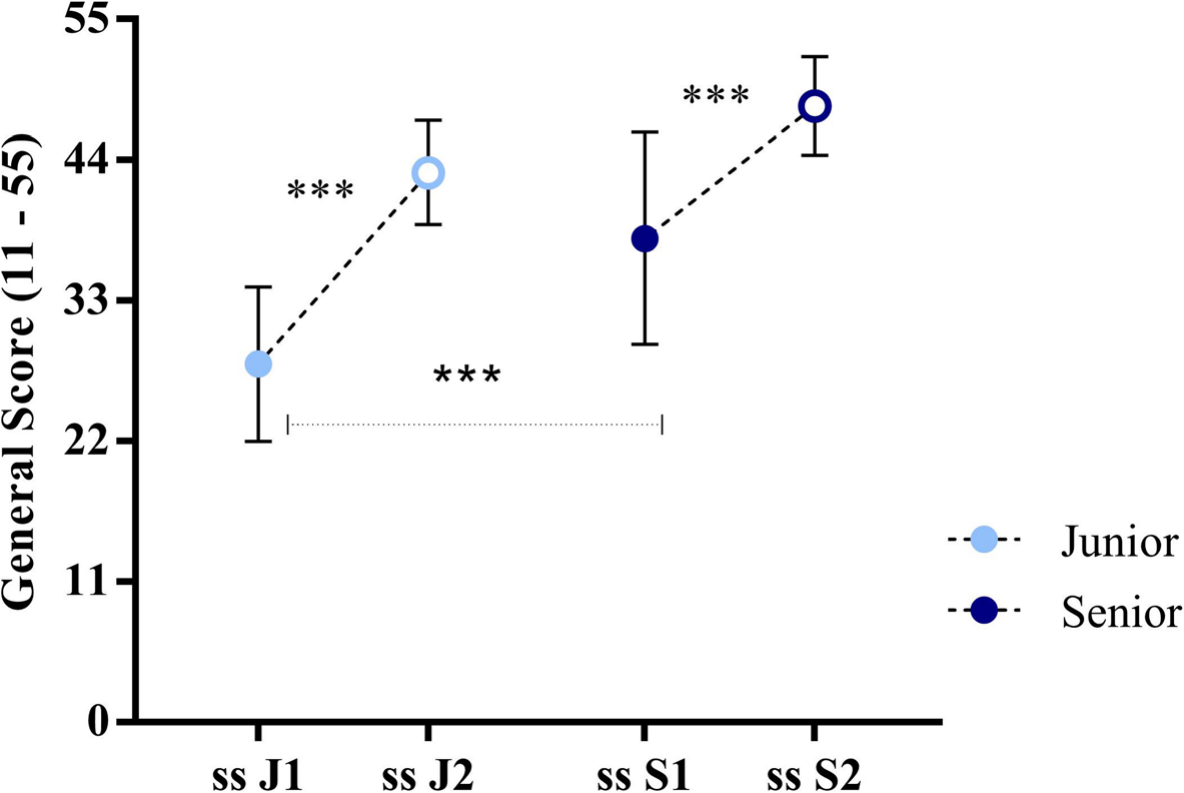
Training with simulation improves technical skills both in junior than senior residents. Residents were divided into two groups: junior (1st, 2nd, 3rd year) and senior (4th, 5th year. Training session 2 was 8 weeks later with the same scenario of session 1. Data are presented as means ± standard deviation and were analyzed statistically by ordinary one-way ANOVA followed by Tukey multiple comparison tests. *p* value < 0.0001(***)

Most of the criticisms about the use of simulation in training are it is useful only in pre-trained trainees. In our study, simulation for OVD significantly improves technical skills both in junior and senior residents. At the beginning of session 1, junior residents presented a total score of 28.01 ± 6.0 and after 8 weeks, they improved to 43.01 ± 4.0. In the same way, senior residents presented a total score of 37.86 ± 8.3 at the beginning of session 1, and they improved their scores to 48.2 ± 3.8 after session 2 (Fig. 5).

### Simulation training significantly modifies self-assessment of trainees

Another aspect of simulation training, highly debated, is the lack of realism. For this reason, before starting the scenario, trainees received a specific questionnaire to evaluate their self-concern and their management based on their clinical evaluation (Fig. 2).

**Fig. 6:**
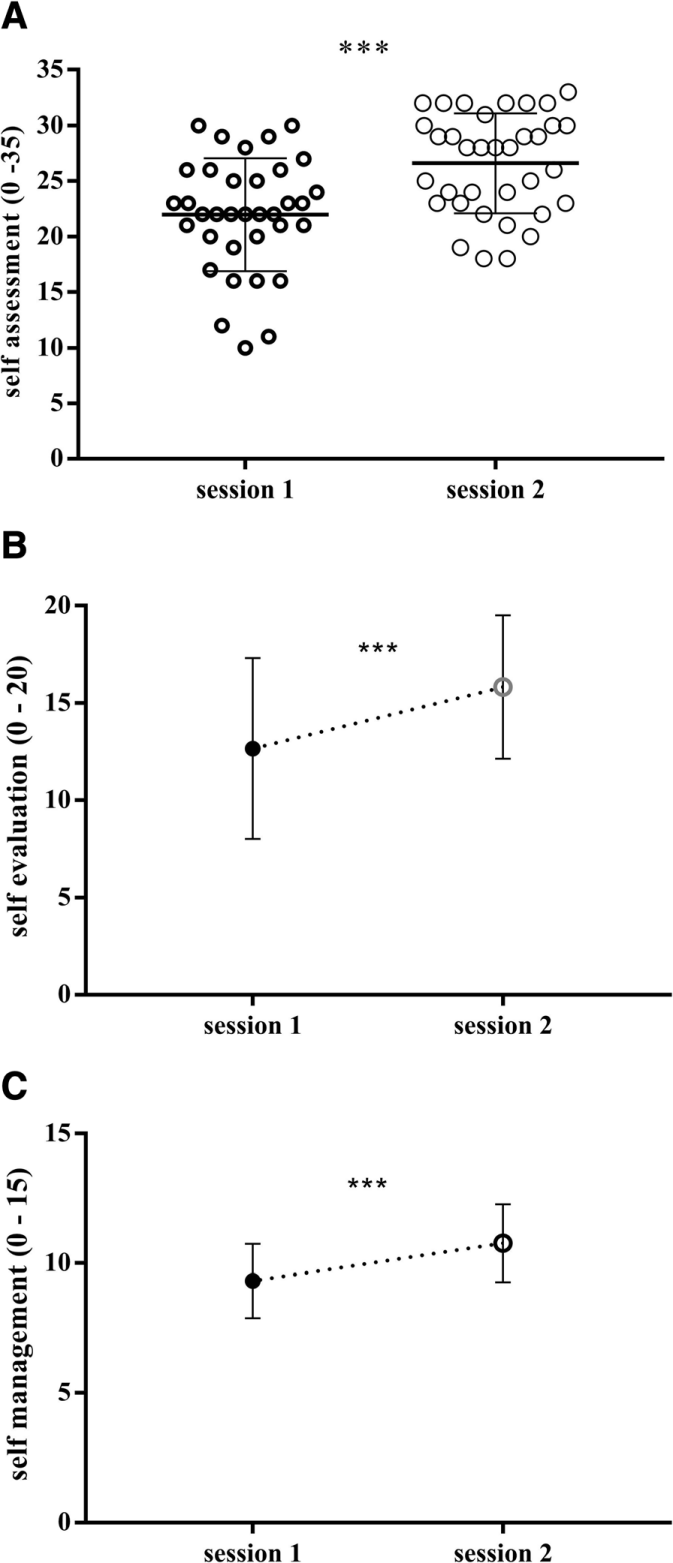
Simulation training significantly modify self-assessment of trainees. Before starting scenario, trainees received a specific questionnaire to evaluate their self-concern and their management based on their clinical evaluation. The evaluation in session 2 was 8 weeks later of session 1. Graph characterize the evaluation of self-assessment (**a**), self-evaluation queries (**b**), and self-management queries (**C**). Data are presented as means ± standard deviation and were analyzed statistically by paired T-test two-tail or Wilcoxon matched-pairs signed rank. *p* value < 0.0001(***)

Before the training start, in session 1, the total score of self-assessment was 21.97 ± 5.1. After the simulation training session, 8 weeks later, the total score was 26.6 ± 4.5 with *p*-value < 0.0001 (Fig. 6a). It is very interesting to note that this improvement was statically significant both in the self-evaluation queries (1–4) (Fig. 6b) than self-management queries (5–7) (Fig. 6c).

## Discussion

In this study, we tested the correct application of the obstetric vacuum called kiwi-cup with a specific assessment scale. We demonstrated that the use of simulation for the application of this instrument is pivotal in the process of learning and acquisition of technical skills. Simulation training plays an important role not only because it provides operators with the technical skills to carry out the application of the kiwi-cup correctly but also and above all because the awareness of having certain skills improves the safety of operators in doing the right things at the right time.

Much has been written in the literature in recent years about strategies to increase the accuracy and safety of OVD [12] with specific attention to simulation [13] [14].

Although there is a general approval on the use of the simulation to improve maternal and fetal outcomes after OVD, there is not a standardized assessment scale for the application of the kiwi-cup. Beyond the successful rate, it is not possible to evaluate the technical skills of the trainee during the application of the kiwi-cup which allows the completion of the birth.

On the other hand, the successful rate does not automatically correspond to a correct application of the kiwi-cup. In clinical practice, this is important because simulation training improves maternal and fetal outcomes even if it does not increase the successful rate [15]. The correct application of the cup limits iatrogenic damage but if the tractions are not performed correctly, it can be detached anyway. Previous papers use the successful rate as a unique and exclusive reference and even in the last large study (STROBE), the primary outcome is to evaluate failed operative vaginal birth with the first intended instrument with no consideration about the used techniques [10]. In a recent systematic review of 30,813 articles about simulation training for operative vaginal delivery, only eight papers evaluated the effects of simulation on technical skills [16]. Among those, only one regarded the application of kiwi-cup and it evaluated an overall “improved knowledge” [17].

It is, therefore, necessary to establish an assessment scale to evaluate whether the operator performs the necessary procedures and whether the improvement of the successful rate (which we also have in our study) does not depend on greater confidence of the operator with the mannequin but rather from a real acquisition of technical skills. At the same time, this evaluation scale allows us to establish exactly which technical skills are lacking and how we can improve the performance of each operator by correcting them.

For this reason, we have idealized an evaluation scale based on the indications directly exposed by Vacca [18].

Among these parameters, the time factor is essential. The duration of OVD is associated with adverse obstetric and neonatal outcomes [19]. We tried to consider even this important element even if, without a doubt, in simulation training, many parameters can differ from the clinical reality: the number of pushes, maternal contractions, psychological pressure, etc. However, in our study, we demonstrated a significant reduction in the duration of operative vaginal delivery.

Also, to make the simulation more likely and to bypass the exclusively technical aspects, a specific scenario was built. Trainees, also bases on their technical skills acquired and beyond their success rates, showed to have a different attitude with the vacuum application procedure. The operator’s experience therefore would not only be important to reduce maternal [20] and neonatal [21] adverse outcomes but also to modulate the decision-making skills of the operators themselves.

This issue constitutes the second original element compared to previous studies. For the first time, the concept of operator experience and, above all, the self-confidence on vacuum application is addressed as a pivotal issue in the decision-making process.

This point of view is innovative compared to literature. Even if the technical skills of each operator play an important role in carrying out the procedures and in doing it with confidence, however, the use of simulation can improve the overall performance even in less experienced operators.

After 8 weeks, the simulation training determines a statistically significant improvement on all the performance of the trainees, including the successful rate and the time taken to complete the procedure.

The choice to evaluate trainees directly at 8 weeks and not after the first training session is not causal. Many studies confirm that the importance of simulation as a type of learning is in the immediate but, above all, in the “knowledge retention” which diminishes with time especially with traditional teaching [17]. In our work, we demonstrate a statistically significant improvement in the technical skills of operators that remains at high levels after about 2 months from the training.

However, our work presents some limitations. For the simulation session, we used a simulator called “Lucy and her Mum”. This model was born with the aim of teaching and allowing to learn the correct application of the kiwi-cup, and it is the same used by Prof. Vacca in his exhibitions. In our humble opinion, it has technical characteristics that partially influence the pelvic-perineal evaluation, which is instead possible in other models. Even the same quantity of lubricant used can interfere with the correct application and the successful rate. These elements are well known for those who do simulation and can be easily overcome in a common training session. However, in our case, since we used a very accurate evaluation scale, data may only partly be affected by these small details. For this reason, it would be desirable that this evaluation scale could be validated with other types of mannequins and in a different scenario to truly develop a standardized evaluation scale beyond the system used.

In conclusion, our work supports the use of simulation for learning the correct procedures for applying kiwi-cup. Besides, it provides a significant step towards introducing specific scales of evaluation in training sessions to standardize the assessment of individual technical skills of trainees.

## Data Availability

All data generated or analyzed during this study are included in this published article.
